# A note on a difference-type estimator for population mean under two-phase sampling design

**DOI:** 10.1186/s40064-016-2368-1

**Published:** 2016-06-14

**Authors:** Mursala Khan, Abdullah Yahia Al-Hossain

**Affiliations:** Department of Mathematics, COMSATS Institute of Information Technology, Abbottabad, 22060 Pakistan; Department of Mathematics, Faculty of Science, Jazan University, Jazan, 2097 Saudi Arabia

**Keywords:** Study variable, Auxiliary variable, Bias, Mean squared-error, Two phase sampling, Exponential chain-type estimator, Efficiency

## Abstract

In this manuscript, we have proposed a difference-type estimator for population mean under two-phase sampling scheme using two auxiliary variables. The properties and the mean square error of the proposed estimator are derived up to first order of approximation; we have also found some efficiency comparison conditions for the proposed estimator in comparison with the other existing estimators under which the proposed estimator performed better than the other relevant existing estimators. We show that the proposed estimator is more efficient than other available estimators under the two phase sampling scheme for this one example; however, further study is needed to establish the superiority of the proposed estimator for other populations.

## Background

In survey sampling, the use of the auxiliary information at the estimation stage is widely used in order to obtain improved designs and the precision of an estimator of the unknown population parameter. When the knowledge of the auxiliary variable is used at the estimation stage, the ratio, product and regression methods of estimation are widely employed in these situations.

The most important topic which is widely discussed in the various probability sampling schemes is the estimation of the population mean of the study variable. A large number of authors have paid their attention towards the formulation of new or modified estimators for the estimation of population mean, for the case, see Hansen and Hurwitz (1943), Sukhatme (1962), Srivastava (1970), Chand (1975), Cochran (1977), Kiregyera (1980, 1984), Srivastava et al. (1990), Bahl and Tuteja (1991), Singh et al. (2006, 2007, 2011), Singh and Choudhury (2012), Khare et al. (2013), Singh and Majhi (2014) and Khan (2015, 2016) etc.

## Symbols and notations

Let us consider a finite population of size *N* of different units *U* = {*U*_1_, *U*_2_, *U*_3_, …, *U*_*N*_}. Let *y* and *x* be the study and the auxiliary variable with corresponding values *y*_*i*_ and *x*_*i*_ respectively for the *i*-th unit *i* = {1, 2, 3,…, *N*} defined in a finite population *U* with means $\bar{Y} = (1/N)\sum\nolimits_{i = 1}^{N} {y_{i} }$ and $\bar{X} = \left( {{1 \mathord{\left/ {\vphantom {1 N}} \right. \kern-0pt} N}} \right)\sum\nolimits_{i = 1}^{N} {x_{i} }$ of the study as well as auxiliary variable respectively.

Also let $S_{y}^{2} = \left( {{1 \mathord{\left/ {\vphantom {1 {N - 1}}} \right. \kern-0pt} {N - 1}}} \right)\sum\nolimits_{i = 1}^{N} {\left( {y_{i} - \bar{Y}} \right)^{2} }$ and $S_{x}^{2} = \left( {{1 \mathord{\left/ {\vphantom {1 {N - 1}}} \right. \kern-0pt} {N - 1}}} \right)\sum\nolimits_{i = 1}^{N} {\left( {x_{i} - \bar{X}} \right)^{2} }$ be the population variances of the study and the auxiliary variable respectively and let *C*_*y*_ and *C*_*x*_ be the coefficient of variation of the study as well as auxiliary variable respectively, and *ρ*_*yx*_ be the correlation coefficient between *x* and *y*. Let *y* and *x* be the study and the auxiliary variable in the sample with corresponding values *y*_*i*_ and *x*_*i*_ respectively for the *i*-th unit *i* = {1, 2, 3…, *n*} in the sample with unbiased means $\bar{y} = \left( {{1 \mathord{\left/ {\vphantom {1 n}} \right. \kern-0pt} n}} \right)\sum\nolimits_{i = 1}^{n} {y_{i} }$ and $\bar{x} = \left( {{1 \mathord{\left/ {\vphantom {1 n}} \right. \kern-0pt} n}} \right)\sum\nolimits_{i = 1}^{n} {x_{i} }$ respectively.

Also let $\hat{S}_{y}^{2} = \left( {{1 \mathord{\left/ {\vphantom {1 {n - 1}}} \right. \kern-0pt} {n - 1}}} \right)\sum\nolimits_{i = 1}^{n} {\left( {y_{i} - \bar{y}} \right)^{2} }$ and $\hat{S}_{x}^{2} = \left( {{1 \mathord{\left/ {\vphantom {1 {n - 1}}} \right. \kern-0pt} {n - 1}}} \right)\sum\nolimits_{i = 1}^{n} {\left( {x_{i} - \bar{x}} \right)^{2} }$ be the corresponding sample variances of the study as well as auxiliary variable respectively. Let $S_{yx} = \frac{{\sum\nolimits_{i = 1}^{N} {\left( {y_{i} - \bar{Y}} \right)\left( {x_{i} - \bar{X}} \right)} }}{N - 1},S_{yz} = \frac{{\sum\nolimits_{i = 1}^{N} {\left( {y_{i} - \bar{Y}} \right)\left( {z_{i} - \bar{Z}} \right)} }}{N - 1}$ and $S_{xz} = \frac{{\sum\nolimits_{i = 1}^{N} {\left( {x_{i} - \bar{X}} \right)\left( {z_{i} - \bar{Z}} \right)} }}{N - 1}$ be the co-variances between their respective subscripts respectively. Similarly $b_{yx\left( n \right)} = \frac{{\hat{S}_{yx} }}{{\hat{S}_{x}^{2} }}$ is the corresponding sample regression coefficient of *y* on *x* based on a sample of size *n*. Also $C_{y} = \frac{{S_{y} }}{{\bar{Y}}},\quad C_{x} = \frac{{S_{x} }}{{\bar{X}}}$ and $C_{z} = \frac{{S_{z} }}{{\bar{Z}}}$ are the coefficients of variations of the study and auxiliary variables respectively.

Also $$\theta = \left( {\frac{1}{n} - \frac{1}{N}} \right),\;\;\theta_{1} = \left( {\frac{1}{{n^{\prime}}} - \frac{1}{N}} \right)$$ and $$\theta_{2} = \left( {\frac{1}{n} - \frac{1}{{n^{\prime}}}} \right).$$

## Some existing estimators

Let us consider a finite population *U* = {*U*_1_, *U*_2_, *U*_3_, …, *U*_*N*_} of size *N* units. To estimate the population mean $\bar{Y}$ of the variable of interest say *y* taking values *y*_*i*_, in the existence of two auxiliary variables say *x* and *z* taking values *x*_*i*_ and *z*_*i*_ for the *i*th unit *U*_*i*_. We assume that there is a high correlation between *y* and *x* as compared to the correlation between *y* and *z*, (i.e. *ρ*_*yx*_ > *ρ*_*yz*_ > 0). When the population $\bar{X}$ of the auxiliary variable *x* is unknown, but information on the other cheaply auxiliary variable say *z* closely related to *x* but compared to *x* remotely to *y*, is available for all the units in a population. In such a situation we use a two phase sampling. In the two phase sampling scheme a large initial sample of size *n′* (*n′* < *N*) is drawn from the population *U* by using simple random sample without replacement sampling (SRSWOR) scheme and measure *x* and *z* to estimate $\bar{X}$. In the second phase, we draw a sample (subsample) of size *n* from first phase sample of size *n*′, i.e. (*n* < *n*′) by using (SRSWOR) or directly from the population *U* and observed the study variable *y*.

The variance of the usual simple estimator $t_{0} = \bar{y} = \frac{1}{n}\sum\nolimits_{i = 1}^{n} {y_{i} }$ up to first order of approximation is, given by

1 $$V\left( {t_{0} } \right) = \theta S_{y}^{2}$$

The classical ratio and regression estimators in two-phase probability sampling and their mean square errors up to first order of approximation are, given by

2 $$t_{1} = \frac{{\bar{y}}}{{\bar{x}}}\bar{x}^{\prime}$$

3 $$MSE\left( {t_{1} } \right) = \bar{Y}^{2} \left[ {\theta C_{y}^{2} + \theta_{2} \left( {C_{x}^{2} - 2\rho_{yx} C_{y} C_{x} } \right)} \right]$$

4 $$t_{2} = \bar{y} + b_{yx\left( n \right)} \left( {\bar{x}^{\prime} - \bar{x}} \right)$$

5 $$MSE\left( {t_{2} } \right) = S_{y}^{2} \left[ {\theta \left( {1 - \rho_{yx}^{2} } \right) + \theta_{1} \rho_{yx}^{2} } \right]$$

Chand (1975), suggested the following chain ratio-type estimator the suggested estimator is, given by

6 $$t_{3} = \frac{{\bar{y}}}{{\bar{x}}}\frac{{\bar{x}^{\prime}}}{{\bar{z}^{\prime}}}\bar{Z}$$

The mean square error of the suggested estimator is, given as

7 $$MSE\left( {t_{3} } \right) = \bar{Y}^{2} \left[ {\theta C_{y}^{2} + \theta_{2} \left( {C_{x}^{2} - 2\rho_{yx} C_{y} C_{x} } \right) + \theta_{1} \left( {C_{z}^{2} - 2\rho_{yz} C_{y} C_{z} } \right)} \right]$$

Khare et al. (2013), proposed a generalized chain ratio in regression estimator for population mean, the recommended estimator is given by

8 $$t_{4} = \bar{y} + b_{yx} \left\{ {\bar{x}^{\prime}\left( {\frac{{\bar{Z}}}{{\bar{z}^{\prime}}}} \right)^{\alpha } - \bar{x}} \right\}$$

where *α* is the unknown constant, and the minimum mean square error at the optimum value of $\alpha = \frac{{\rho_{yz} C_{x} }}{{\rho_{yx} c_{z} }}$ is, given by

9 $$MSE\left( {t_{4} } \right) = \bar{Y}^{2} C_{y}^{2} \left[ {\theta - \theta_{2} \rho_{yx}^{2} - \theta_{1} \rho_{yz}^{2} } \right]$$

Recently Singh and Mahji (2014), suggested a chain-type exponential estimators for $\bar{Y}$ given by

10 $$t_{5} = \frac{{\bar{y}}}{{\bar{x}}}\bar{x}^{\prime}\exp \left( {\frac{{\bar{Z} - \bar{z}^{\prime}}}{{\bar{Z} + \bar{z}^{\prime}}}} \right)$$

11 $$t_{6} = \bar{y} + b_{yx\left( n \right)} \left\{ {\bar{x}^{\prime}\exp \left( {\frac{{\bar{Z} - \bar{z}^{\prime}}}{{\bar{Z} + \bar{z}^{\prime}}}} \right) - \bar{x}} \right\}$$

12 $$t_{7} = \bar{y}\exp \left( {\frac{{\bar{x}^{\prime} - \bar{x}}}{{\bar{x}^{\prime} + \bar{x}}}} \right)\frac{{\bar{Z}}}{{\bar{z}^{\prime}}}$$

The mean square errors of the suggested estimators, up to first order of approximation are, given as follows

13 $$MSE\left( {t_{5} } \right) = \bar{Y}^{2} \left[ {\theta C_{y}^{2} + \theta_{2} \left( {C_{x}^{2} - 2\rho_{yx} C_{y} C_{x} } \right) + \frac{{\theta_{1} }}{4}\left( {C_{z}^{2} - 4\rho_{yz} C_{y} C_{z} } \right)} \right]$$

14 $$MSE\left( {t_{6} } \right) = \bar{Y}^{2} C_{y}^{2} \left[ {\theta_{2} \left( {1 - \rho_{yx}^{2} } \right) + \theta_{1} \left( {1 + \frac{{\rho_{yx}^{2} }}{4}\frac{{C_{z}^{2} }}{{C_{x}^{2} }} - \rho_{yx} \rho_{yz} \frac{{C_{z} }}{{C_{x} }}} \right)} \right]$$

15 $$MSE\left( {t_{7} } \right) = \bar{Y}^{2} \left[ {\theta C_{y}^{2} + \frac{{\theta_{2} }}{4}\left( {C_{x}^{2} - 4\rho_{yx} C_{y} C_{x} } \right) + \theta_{1} \left( {C_{z}^{2} - 2\rho_{yz} C_{y} C_{z} } \right)} \right]$$

## The proposed estimator

On the lines of Khare et al. (2013), we propose a difference-type estimator for population mean under two-phase sampling scheme using two auxiliary variables; the suggested estimator is, given by

16 $$t_{m} = \bar{y} + k_{1} \left( {\bar{x}^{\prime}\frac{{\bar{Z}}}{{\bar{z}^{\prime}}} - \bar{x}} \right) + k_{2} \left( {\bar{Z}\frac{{\bar{x}^{\prime}}}{{\bar{x}}} - \bar{z}} \right)$$

where *k*_1_ and *k*_2_ are the unknown constants,

To obtain the properties of the proposed estimator we define the following relative error terms and their expectations.

Let $e_{0} = \frac{{\bar{y} - \bar{Y}}}{{\bar{Y}}},\;e_{1} = \frac{{\bar{x} - \bar{X}}}{{\bar{X}}},\;e^{\prime}_{1} = \frac{{\bar{x}^{\prime} - \bar{X}}}{{\bar{X}}},\;e_{2} = \frac{{\bar{z} - \bar{Z}}}{{\bar{Z}}}$ and $e^{\prime}_{2} = \frac{{\bar{z} - \bar{Z}}}{{\bar{Z}}}$, such that

$$\begin{aligned} E\left( {e_{0} } \right) &= E\left( {e_{i} } \right) = E\left( {e_{i}^{{\prime }} } \right) = 0, \quad {\text{for}}\;i = 1, \, 2.\\ E\left( {e_{0}^{2} } \right) & = \theta C_{y}^{2} ,E\left( {e_{1}^{2} } \right) = \theta C_{x}^{2} ,E\left( {e_{1}^{\prime 2} } \right) = \theta_{1} C_{x}^{2} ,E\left( {e_{1} e^{\prime }_{1} } \right) = \theta_{1} C_{x}^{2} , E\left( {e_{2}^{2} } \right) = \theta C_{z}^{2} , \\ E\left( {e_{0} e^{\prime }_{2} } \right) & = \theta_{1} C_{yz} , E\left( {e_{0} e_{1} } \right) = \theta C_{yx} ,E\left( {e_{0} e^{\prime }_{1} } \right) = \theta_{1} C_{yx} , E\left( {e_{0} e_{2} } \right) = \theta C_{yz} , \\ E\left( {e_{1} e_{2}^{\prime } } \right) & = E\left( {e_{1}^{\prime } e_{2}^{\prime } } \right) = \theta_{1} C_{xz} , E\left( {e_{1} e_{2} } \right) = \theta C_{xz} , E\left( {e_{2}^{\prime 2} } \right) = E\left( {e_{2} e_{2}^{\prime } } \right) = \theta_{1} C_{z}^{2} . \\ \end{aligned}$$

Rewriting (16), in terms of *e*’*s*, we have

$$t_{m} = \left[ {\bar{Y}\left( {1 + e_{0} } \right) + k_{1} \bar{X}\left( {\left( {1 + e_{1}^{{\prime }} } \right)\left( {1 + e_{2}^{{\prime }} } \right)^{ - 1} - \left( {1 + e_{1} } \right)} \right) + k_{2} \bar{Z}\left( {\left( {1 + e_{1}^{{\prime }} } \right)\left( {1 + e_{1} } \right)^{ - 1} - \left( {1 + e_{2} } \right)} \right)} \right]$$

Expanding the right hand side of the above equation, and neglecting terms of *e*’*s* having power greater than two, we have

17 $$t_{m} - \bar{Y} = \left[ {\bar{Y}e_{0} - k_{1} \bar{X}\left( {e_{1} - e_{1}^{{\prime }} + e_{2}^{{\prime }} + e_{2}^{{{\prime 2}}} + e_{1}^{{\prime }} e_{2}^{{\prime }} } \right) - k_{2} \bar{Z}\left( {e_{1} - e_{1}^{{\prime }} + e_{2} - e_{1}^{\text{2}} + e_{1} e_{1}^{{\prime }} } \right)} \right]$$

On squaring and taking expectation on both sides of Eq. (17), and keeping terms up to second order, we have

$$\begin{aligned} MSE\left( {t_{m} } \right) & = E\left[ {\bar{Y}^{2} e_{0}^{2} + k_{1}^{2} \bar{X}^{2} \left( {e_{1}^{2} + e_{1}^{{\prime 2}} + e_{2}^{{\prime 2}} - 2e_{1} e_{1}^{\prime } + 2e_{1} e_{2}^{\prime } - 2e_{1}^{\prime } e_{2}^{\prime } } \right)} \right. \\ & \quad + k_{2}^{2} \bar{Z}^{2} \left( {e_{1}^{2} + e_{1}^{{\prime 2}} + e_{2}^{2} - 2e_{1} e_{1}^{\prime } + 2e_{1} e_{2} - 2e_{1}^{\prime } e_{2} } \right) \\ & \quad + 2k_{1} k_{2} \bar{X}\bar{Z}\left( {e_{1}^{2} + e_{1}^{{\prime 2}} - 2e_{1} e_{1}^{\prime } + e_{1} e_{2} - e_{1}^{\prime } e_{2} + e_{2}^{\prime } e_{1} - e_{1}^{{\prime 2}} e_{1}^{\prime } + e_{1}^{\prime } e_{2} } \right) \\ & \left. \quad - 2k_{1} \bar{Y}\bar{X}\left( {e_{0} e_{1} - e_{0} e_{1}^{\prime } + e_{0} e_{2}^{\prime } } \right) - 2k_{2} \bar{Y}\bar{Z}\left( {e_{0} e_{1} - e_{0} e_{1}^{\prime } + e_{0} e_{2} } \right) \right] \\ \end{aligned}$$

Further simplifying, we get

18 $$\begin{aligned} MSE\left( {t_{m} } \right) & = \left[ {\bar{Y}^{2} \theta C_{y}^{2} + k_{1}^{2} \bar{X}^{2} \left( {\theta_{1} C_{z}^{2} + \theta_{2} C_{x}^{2} } \right) + k_{2}^{2} \bar{Z}^{2} \left( {\theta C_{z}^{2} + \theta_{2} C_{x}^{2} + 2\theta_{2} C_{xz} } \right)} \right. \\ & \quad \left. + 2k_{1} k_{2} \bar{X}\bar{Z}\left( {\theta_{2} C_{x}^{2} + \theta_{1} C_{z}^{2} + \theta_{2} C_{xz} } \right) - 2k_{1} \bar{Y}\bar{X}\left( {\theta_{2} C_{yx} + \theta_{1} C_{yz} } \right) - 2k_{2} \bar{Y}\bar{Z}\left( {\theta_{2} C_{yx} + \theta C_{yz} } \right) \right] \\ \end{aligned}$$

Now to find the minimum mean squared error of *t*_*m*_, we differentiate Eq. (18) with respect to *k*_1_ and *k*_2_ respectively and putting it equal to zero, that is

$$\frac{{\partial MSE\left( {t_{m} } \right)}}{{\partial k_{1} }} = 0\quad{\text{and}}\quad\frac{{\partial MSE\left( {t_{m} } \right)}}{{\partial k_{2} }} = 0$$

$$k_{1opt} = \frac{{\bar{Y}\left( {BC - DE} \right)}}{{\bar{X}\left( {AB - E^{2} } \right)}}\quad{\text{and}}\quad k_{2opt} = \frac{{\bar{Y}\left( {AD - CE} \right)}}{{\bar{Z}\left( {AB - E^{2} } \right)}}.$$

where *A* = *θ*_1_*C*_*z*_^2^ + *θ*_2_*C*_*x*_^2^, *B* = *θC*_*z*_^2^ + *θ*_2_*C*_*x*_^2^ + 2*θ*_2_*C*_*xz*_, *C* = *θ*_2_*C*_*yx*_ + *θ*_1_*C*_*yz*_, *D* = *θ*_2_*C*_*yx*_ + *θC*_*yz*_ and *E* = *θ*_1_*C*_*z*_^2^ + *θ*_2_*C*_*x*_^2^ + *θ*_2_*C*_*xz*_.

On substituting the optimum values of *k*_1_ and *k*_2_ in Eq. (18) we get the minimum mean square error (*MSE*) of the proposed estimator *t*_*m*_ up to order one is, given as

19 $$MSE\left( {t_{m} } \right)_{\hbox{min} } = \bar{Y}^{2} \left[ {\theta C_{y}^{2} - \frac{{\left( {AD^{2} + BC^{2} - 2CDE} \right)}}{{\left( {AB - E^{2} } \right)}}} \right]$$

## Efficiency comparison

In this section, we have compare the propose estimator with the other existing estimators.

1. By Eqs. (1) and (19),
  $$MSE\left( {t_{m} } \right)_{\hbox{min} } < MSE\left( {t_{0} } \right)\quad {\text{if}}\quad \left[ {\frac{{\left( {AD^{2} + BC^{2} - 2CDE} \right)}}{{\left( {AB - E^{2} } \right)}}} \right] > 0.$$
2. By Eqs. (3) and (19)
  $$MSE\left( {t_{m} } \right)_{\hbox{min} } < MSE\left( {t_{1} } \right)\quad {\text{if}}\quad \left[ {\frac{{\left( {AD^{2} + BC^{2} - 2CDE} \right)}}{{\left( {AB - E^{2} } \right)}} + \theta_{2} \left( {C_{x}^{2} - 2\rho_{yx} C_{y} C_{x} } \right)} \right] > 0.$$
3. By Eqs. (5) and (19),
  $$MSE\left( {t_{m} } \right)_{\hbox{min} } < MSE\left( {t_{2} } \right)\quad {\text{if}}\quad \left[ {\frac{{\left( {AD^{2} + BC^{2} - 2CDE} \right)}}{{\left( {AB - E^{2} } \right)}} - \theta_{2} C_{y}^{2} \rho_{yx}^{2} } \right] > 0.$$
4. By Eqs. (7) and (19),
  $$MSE\left( {t_{m} } \right)_{\hbox{min} } < MSE\left( {t_{3} } \right)\quad {\text{if}}\quad \left[ {\theta_{2} C_{x} \left( {C_{x} - 2\rho_{yx} C_{y} } \right) + \theta_{1} C_{z} \left( {C_{z} - 2\rho_{yz} C_{y} } \right) + \frac{{\left( {AD^{2} + BC^{2} - 2CDE} \right)}}{{\left( {AB - E^{2} } \right)}}} \right] > 0.$$
5. By Eqs. (9) and (19),
  $$MSE\left( {t_{m} } \right)_{\hbox{min} } < MSE\left( {t_{4} } \right)\quad {\text{if}}\quad \left[ {\frac{{\left( {AD^{2} + BC^{2} - 2CDE} \right)}}{{\left( {AB - E^{2} } \right)}} - \left( {\theta_{2} \rho_{yx}^{2} + \theta_{1} \rho_{yz}^{2} } \right)C_{y}^{2} } \right] > 0.$$
6. By Eqs. (13) and (19),
  $$MSE\left( {t_{m} } \right)_{\hbox{min} } < MSE\left( {t_{5} } \right)\quad {\text{if}}\quad \left[ {\theta_{2} \left( {C_{x}^{2} - 2C_{xy} } \right) + \frac{{\theta_{1} }}{4}\left( {C_{z}^{2} - 4C_{yz} } \right) + \frac{{\left( {AD^{2} + BC^{2} - 2CDE} \right)}}{{\left( {AB - E^{2} } \right)}}} \right] > 0.$$
7. By Eqs. (14) and (19),
  $$MSE\left( {t_{m} } \right)_{\hbox{min} } < MSE\left( {t_{6} } \right)\quad {\text{if}}\quad \left[ {\theta_{1} C_{y}^{2} \left( {\frac{{\rho_{yx}^{2} }}{4}\frac{{C_{z}^{2} }}{{C_{x}^{2} }} - \rho_{yx} \rho_{yz} \frac{{C_{z} }}{{C_{x} }}} \right) - \theta_{2} \rho_{yx}^{2} C_{y}^{2} + \frac{{\left( {AD^{2} + BC^{2} - 2CDE} \right)}}{{\left( {AB - E^{2} } \right)}}} \right] > 0.$$
8. By Eqs. (15) and (19),
  $$MSE\left( {t_{m} } \right)_{\hbox{min} } < MSE\left( {t_{7} } \right)\quad {\text{if}}\quad \left[ {\frac{{\left( {AD^{2} + BC^{2} - 2CDE} \right)}}{{\left( {AB - E^{2} } \right)}} + \frac{{\theta_{2} }}{4}\left( {C_{x}^{2} - 4C_{xy} } \right) + \theta_{1} \left( {C_{z}^{2} - 2C_{yz} } \right)} \right] > 0.$$

## Numerical comparison

To examine the performance of the proposed estimator with various existing estimators, we have considered a real data set from the literature the description of the population are, given by

*Population* Source, (Cochran 1977).

*y*: Number of placebo children;

*x*: Number of paralytic polio cases in the placebo group;

*z*: Number of paralytic polio cases in the not inoculated group.

$N = 34,n^{\prime} = 15,n = 10,\bar{Y} = 4.92,\bar{X} = 2.59,\bar{Z} = 2.91,$*C*_*y*_^2^ = 1.0248, *C*_*x*_^2^ = 1.5175, *C*_*z*_^2^ = 1.1492, *C*_*yx*_ = 0.9136, *C*_*yz*_ = 0.6978, *ρ*_*yx*_ = 0.7326, *ρ*_*yz*_ = 0.6430, *ρ*_*xz*_ = 0.6837 (Table 1). We have use the following expression for Percentage Relative Efficiency (*PRE*)

**Table 1:** The mean square errors (*MSE’s*) and the Percent relative efficiencies (*PRE’s*) of the estimators with respect to *t*_0_

Population		
Estimator	*MSE’s*	*PRE* (*t*_0_,*t*_*j*_)
*t*_0_	1.7525	100.00
*t*_1_	1.5032	116.59
*t*_2_	1.3073	134.06
*t*_3_	1.2793	137.00
_*t*4_	0.9247	189.52
*t*_5_	1.1312	154.92
*t*_6_	1.0227	171.36
*t*_7_	1.0982	159.58
*t*_*m*_	0.8206	213.56

$$PRE = \left[ {\frac{{Var(t_{0} )}}{{MSE\left( {t_{j} } \right)\;or\;Var(t_{j} )}}} \right] * 100,\quad {\text{for}}\;j = 0, \, 1, \, 2, \, 3, \, 4, \, 5, \, 6,7\;{\text{and}}\;m.$$

## Conclusion

From the above table, we have observed that the proposed estimator has smaller mean square error and has higher percent relative efficiency than the other existing estimators. However, although the proposed estimator has the highest percent relative efficiency than other existing estimators for this one example, it could have lower relative efficiency for other populations. Further work is needed before it can be recommended for general use in practical surveys.
